# Genomic monitoring of SARS‐CoV‐2 variants using sentinel SARI hospital surveillance

**DOI:** 10.1111/irv.13202

**Published:** 2023-10-13

**Authors:** Sarah Denayer, François E. Dufrasne, Bert Monsieurs, Reinout van Eycken, Sarah Houben, Lucie Seyler, Thomas Demuyser, Els van Nedervelde, Marc Bourgeois, Bénédicte Delaere, Koen Magerman, Door Jouck, Bénédicte Lissoir, Catherine Sion, Marijke Reynders, Evelyn Petit, Nicolas Dauby, Marc Hainaut, Lies Laenen, Piet Maes, Guy Baele, Simon Dellicour, Lize Cuypers, Emmanuel André, Simon Couvreur, Ruben Brondeel, Cyril Barbezange, Nathalie Bossuyt, Steven van Gucht

**Affiliations:** ^1^ Viral Diseases, National Influenza Centre, Scientific Directorate of Infectious Diseases in Humans Sciensano Ukkel Belgium; ^2^ Observational Clinical Trials, Scientific Directorate of infectious Diseases in Humans Sciensano Ukkel Belgium; ^3^ Department of Internal Medicine and Infectiology, Universitair Ziekenhuis Brussel (UZB) Vrije Universiteit Brussel (VUB) Brussels Belgium; ^4^ Department of Microbiology and Infection Control, Universitair Ziekenhuis Brussel (UZB) Vrije Universiteit Brussel (VUB) Brussels Belgium; ^5^ AIMS Lab, Center for Neurosciences, Faculty of Medicine and Pharmacy Vrije Universiteit Brussel (VUB) Brussels Belgium; ^6^ CHU UCL Namur, site Mont‐Godinne Yvoir Belgium; ^7^ Infection Control and Clinical Laboratory Jessa Ziekenhuis Hasselt Belgium; ^8^ Department of Immunology and Infection Hasselt University Hasselt Belgium; ^9^ Infection Control Jessa Ziekenhuis Hasselt Belgium; ^10^ Laboratory Site St‐Joseph Grand Hôpital de Charleroi Gilly Belgium; ^11^ Laboratory Medicine AZ Sint‐Jan Brugge‐Oostende AV Bruges Belgium; ^12^ Department of Infectious Diseases, Centre Hospitalier Universitaire Saint‐Pierre Université Libre de Bruxelles (ULB) Brussels Belgium; ^13^ Institute for Medical Immunology, ULB Center for Research in Immunology (U‐CRI) Université Libre de Bruxelles (ULB) Brussels Belgium; ^14^ School of Public Health Université Libre de Bruxelles (ULB) Brussels Belgium; ^15^ Pediatrics Department, CHU Saint‐Pierre Université Libre de Bruxelles (ULB) Brussels Belgium; ^16^ National Reference Center for Respiratory Pathogens, UZ Leuven University Hospitals Leuven Leuven Belgium; ^17^ Laboratory of Clinical Microbiology, Department of Microbiology, Immunology and Transplantation KU Leuven Leuven Belgium; ^18^ Department of Microbiology, Immunology and Transplantation, Rega Institute KU Leuven Leuven Belgium; ^19^ Spatial Epidemiology Lab (SpELL) Université Libre de Bruxelles Brussels Belgium; ^20^ Epidemiology and public Health, Epidemiology of Infectious Diseases Sciensano Brussels Belgium

**Keywords:** genomic surveillance, influenza, pandemic preparedness, respiratory viruses, SARI surveillance, SARS‐CoV‐2

## Abstract

**Background:**

To support the COVID‐19 pandemic response, many countries, including Belgium, implemented baseline genomic surveillance (BGS) programs aiming to early detect and characterize new SARS‐CoV‐2 variants. In parallel, Belgium maintained a sentinel network of six hospitals that samples patients with severe acute respiratory infections (SARI) and integrated SARS‐CoV‐2 detection within a broader range of respiratory pathogens. We evaluate the ability of the SARI surveillance to monitor general trends and early signals of viral genetic evolution of SARS‐CoV‐2 and compare it with the BGS as a reference model.

**Methods:**

Nine‐hundred twenty‐five SARS‐CoV‐2 positive samples from patients fulfilling the Belgian SARI definition between January 2020 and December 2022 were sequenced using the ARTIC Network amplicon tiling approach on a MinION platform. Weekly variant of concern (VOC) proportions and types were compared to those that were circulating between 2021 and 2022, using 96,251 sequences of the BGS.

**Results:**

SARI surveillance allowed timely detection of the Omicron (BA.1, BA.2, BA.4, and BA.5) and Delta (B.1.617.2) VOCs, with no to 2 weeks delay according to the start of their epidemic growth in the Belgian population. First detection of VOCs B.1.351 and P.1 took longer, but these remained minor in Belgium. Omicron BA.3 was never detected in SARI surveillance. Timeliness could not be evaluated for B.1.1.7, being already major at the start of the study period.

**Conclusions:**

Genomic surveillance of SARS‐CoV‐2 using SARI sentinel surveillance has proven to accurately reflect VOCs detected in the population and provides a cost‐effective solution for long‐term genomic monitoring of circulating respiratory viruses.

## 1 INTRODUCTION

Since the start of the SARS‐CoV‐2 pandemic in 2019, genomic surveillance to detect emerging variants of concern (VOCs) and variants of interest (VOIs) has been widely used, especially to guide public health actions, initiate early characterization of emerging variants, understand the spatio‐temporal spread of the virus, and understand the impact of emerging mutations on treatment efficacy.^1^, ^2^, ^3^, ^4^, ^5^, ^6^, ^7^, ^8^, ^9^ To date, the World Health Organization (WHO) has designated five VOCs worldwide after the start of the pandemic with the original Wuhan strain.^10^, ^11^, ^12^ The SARS‐CoV‐2 Alpha variant (B.1.1.7) was first identified in the United Kingdom (UK) in late summer to early autumn 2020 and caused a rapid increase in COVID‐19 cases in the UK by the end of 2020.^13^, ^14^ During the same period, a rapid resurgence of the epidemic was caused by the Beta (B.1.351) variant in South Africa,^15^, ^16^, ^17^ and in Brazil, the emergence of a novel VOC, referred to as Gamma (P.1), was reported around early November 2020.^16^ The Delta variant (B.1.617.2), which was first detected in India in January 2021, was designated by the WHO as a VOC in May 2021.^18^ Omicron (B.1.1.529; including lineages BA.1 to BA.5) is the most recently recognized VOC and was first reported by South Africa in November 2021.^19^

During the acute pandemic phase, it has been considered essential to detect, monitor, and assess virus variants that can result in increased transmissibility and disease severity or have other adverse effects on public health and social control measures.^2^, ^20^ To obtain timely and accurate information on the emergence and circulation of VOCs and VOIs, robust surveillance systems, including a well‐defined sampling and sequencing strategy, were required and were implemented in many countries.^21^ It is within this context that the European Center for Disease Prevention and Control (ECDC) recommended two complementary sampling approaches. First, a representative sampling of SARS‐CoV‐2 RT‐PCR positive cases from existing population‐based surveillance systems and, second, a targeted sampling of SARS‐CoV‐2 positive cases occurring in special settings or populations.^2^ In order to comply with these recommendations, Belgium set up a national baseline genomic surveillance (BGS) aiming at sequencing a representative subset (5–10%) of all SARS‐CoV‐2 PCR positive samples in order to follow‐up trends for the circulating viruses and to detect emerging variants when they reach a certain proportion, typically around 1%.^2^, ^22^, ^23^, ^24^, ^25^ Additionally, an active genomic surveillance was also set up to target specific indications, including unusual outbreaks, persisting infections in immunocompromised patients, and returning travelers from a zone at risk.^23^, ^24^, ^26^ Patients hospitalized with severe acute respiratory infections (SARI) were at first not included in these indications, although being a very important group to evaluate the impact of SARS‐CoV‐2 on human health.^27^, ^28^, ^29^, ^30^

Sentinel surveillance networks have been operating for influenza virus for many years.^27^, ^28^, ^29^, ^31^, ^32^ In Belgium, SARI surveillance exists since 2012 and consists presently of a network of six hospitals sending respiratory samples and clinical information from patients fulfilling the case definition to the National Influenza Centre. In particular, SARI surveillance has proven to be very useful to evaluate the severity of infections caused by different respiratory viruses, including influenza virus.^27^, ^28^, ^29^, ^30^, ^33^, ^34^, ^35^, ^36^, ^37^ Our group also demonstrated that non‐influenza respiratory viruses (NIRV) have an important contribution to the burden of SARI, with overall one third of the SARI cases showing positivity for one or more of the respiratory viruses tested (i.e., coronavirus, human metapneumovirus, rhinovirus, enterovirus, and parainfluenza virus or respiratory syncytial virus).^27^, ^28^, ^29^ The information retrieved from the Belgian SARI network system did not only allow to evaluate the severity of these respiratory viruses against the burden of influenza but also to investigate the vaccine efficacy and epidemiology of these viruses. Additionally, the provided data were considered to be beneficial for clinical management of SARI patients.^27^, ^28^, ^29^

In this study, we evaluated the performance of a SARI‐based SARS‐CoV‐2 genomic surveillance for the five different VOCs based on the Belgian sentinel hospital network during the pandemic. This evaluation was performed based on different criteria, namely, the ability to correctly follow major trends obtained from the exhaustive baseline genomic surveillance, the ability to promptly detect major introduction events, and the ability to detect minority variant populations.

After more than 2 years of crisis due to the SARS‐CoV‐2 pandemic, and with many countries now entering the recovery phase and planning for long‐term surveillance of respiratory pathogens, we demonstrate here that the SARI surveillance network is an appropriate and cost‐effective tool to monitor the circulation of SARS‐CoV‐2 variants or variants of other respiratory pathogens.

## 2 METHODS

**FIGURE 1:**
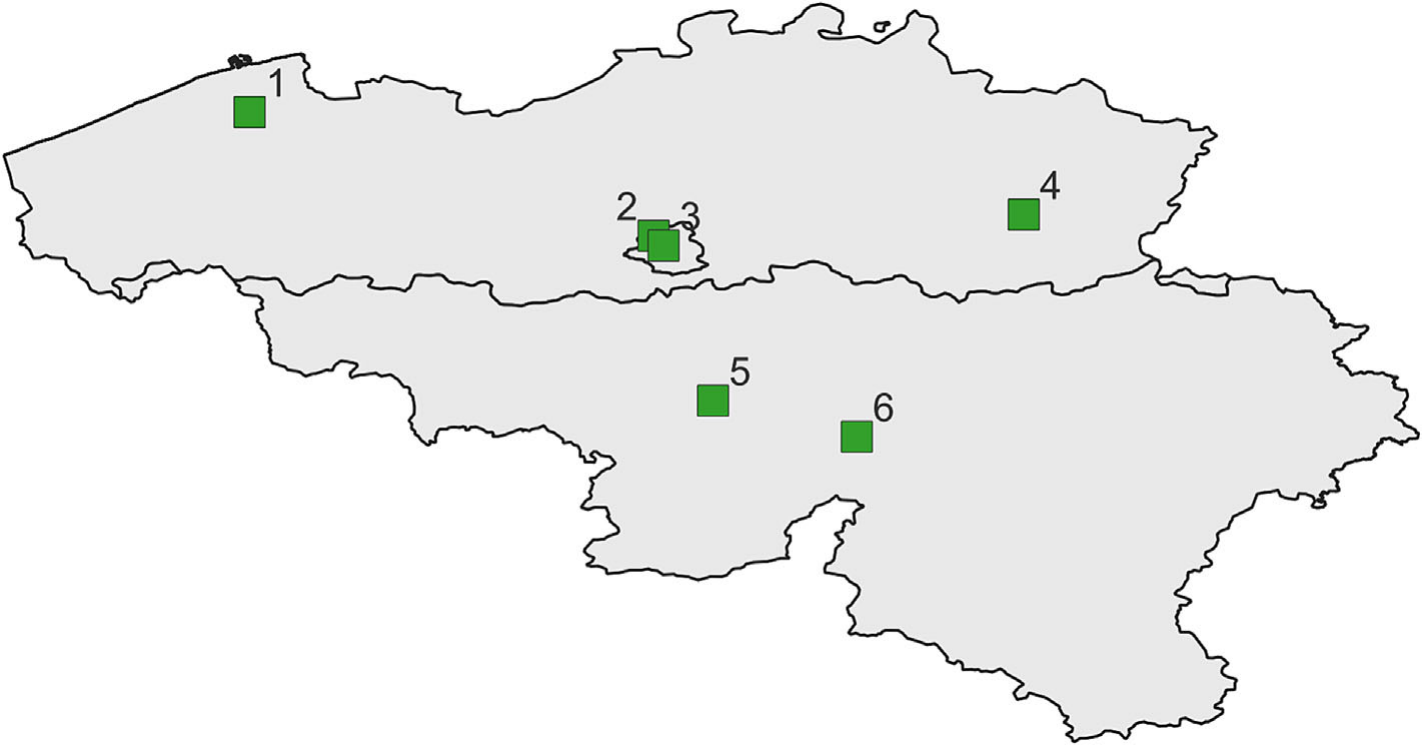
Spatial location of the six hospitals from the Belgian hospital‐based SARI surveillance network (BELSARI‐net). (1) AZ Sint Jan Brugge‐Oostende (site Brugge); (2) Universitair Ziekenhuis Brussel; (3) CHU Saint‐Pierre Brussel; (4) Jessa Ziekenhuis Hasselt; (5) GHdC Charleroi; (6) CHU UCL Namur (site Godinne) (map created using the Free and Open Source QGIS, version 3.22.5).

Belgian sentinel SARI surveillance has been in place since 2012 and is composed of six hospitals spread over the whole country (Figure 1, map created using the Free and Open Source QGIS, version 3.22.5).^27^, ^28^, ^29^ The overall catchment population of the current network is estimated at 992,310 inhabitants, which corresponds to 8.6% of the Belgian population.

At these hospitals, respiratory samples (nasopharyngeal swabs, aspirates, or broncho‐alveolar lavages) were taken from adults and children fulfilling the Belgian SARI definition (adapted from the WHO 2014 SARI case definition). A SARI case is defined as a person suffering from an acute respiratory illness with onset within the last 10 days of (1) history of fever or measured fever of ≥38°C, (2) cough or dyspnea, (3) and requiring hospitalization (24 h or more, at least an overnight stay). Samples were sent to the National Influenza Centre (NIC) hosted by the National Institute of Public Health (Sciensano) in Belgium, where they were tested by RT‐qPCR to detect seasonal influenza viruses (Subtypes A and B), 16 other respiratory viruses and SARS‐CoV‐2.^29^

Whole Genome Sequencing (WGS) of SARS‐CoV‐2 was performed on positive samples taken between 2020 and 2022, and with sufficient high viral load (Ct ≤ 25) on a MinION platform (Oxford Nanopore Technologies) following the ARTIC Network protocol v3.17 or Midnight 1200 bp primer panels (both IDT Technologies) as described previously.^38^, ^39^ cDNA synthesis was performed on RNA extracts (EMAG®, BioMérieux) using the LunaScript® RT SuperMix kit (New England Biolabs). After the PCR, amplicons of each sample were pooled and we used the NEBNext® Ultra II End Repair/dA‐Tailing Module (New England Biolabs) to convert the fragmented DNA to repaired DNA having 5′ phosphorylated ends. Then, repaired amplicons were barcoded with the Native Barcoding Expansion 96 kit (EXP‐NBD196, Oxford Nanopore Technologies) according to the manufacturer's protocol. Barcoded samples were pooled in one tube, bead‐purified (ProNex® Size‐Selective Purification system, Promega), and eluted. After addition of the sequencing adapters (AMII from Oxford Nanopore Technologies) at the ends of the barcoded amplicons, the library was subsequently prepared for sequencing by adding loading beads and finally loaded into a primed R9.4.1 flow cell (Oxford Nanopore Technologies). Complete sequences were recovered using the ARTIC analysis pipeline, and clades and lineages were determined using the open web application Nextclade provided by Nextstrain.^40^ Sequences with a bad QC score in Nextclade were excluded from the analysis. Samples with lower viral load (Ct > 25) or for which insufficient material was available were excluded from the study.

Numbers of identified VOCs in SARI surveillance were calculated per week according to the sampling date and compared to the weekly SARS‐CoV‐2 VOCs circulating in the general population using the baseline genomic surveillance data that are publically available at the Belgium COVID‐19 Dashboard.^41^ The 14‐day forward moving average for variant data (Belgium COVID‐19 Dashboard, accessed 13/06/2023) was kindly transformed into reported numbers per ISO‐week by Sciensano. The concerned study period runs from the official start of the genomic baseline surveillance at week 7 of 2021 (2021‐W07) until 2022‐W52. The timeliness and sensitivity for VOC detection using SARI surveillance were evaluated by comparing the week of the first detection for each VOC within the context of its emergence and its maximal weekly detection level (%) in the baseline genome surveillance, respectively. Weekly detection levels (%) for each VOC were extracted from the baseline genomic surveillance data by dividing the number of sequences reported for a specific VOC by the total number of sequences reported for the same week. ISO weeks were used to perform this calculation. We defined the epidemic growth phase of a VOC as the period for which the exponential increase of its weekly reported proportion (%) is a constant during at least 4 weeks in the BGS, which was calculated using a linear regression curve.^42^, ^43^, ^44^ The slope value *a* of the linear regression curve (equation format y = *a*x + b) within that time window represents the relationship between the detection levels (%) of each VOC in relation with time, and thus provides a measure of how quickly a VOC is growing. The *R* ^2^ value was used as a measure of the quality of the linear regression curve for the used dataset. The weeks between the first detection of a VOC until the start of the epidemic growth phase in the BGS were defined as the low detection phase of this VOC (see Supporting Information S2). Data were processed using Excel 2016 and R 4.2.3 (RStudio Version 2023.06.0).

## 3 RESULTS

### 3.1 General results and observations

**FIGURE 2:**
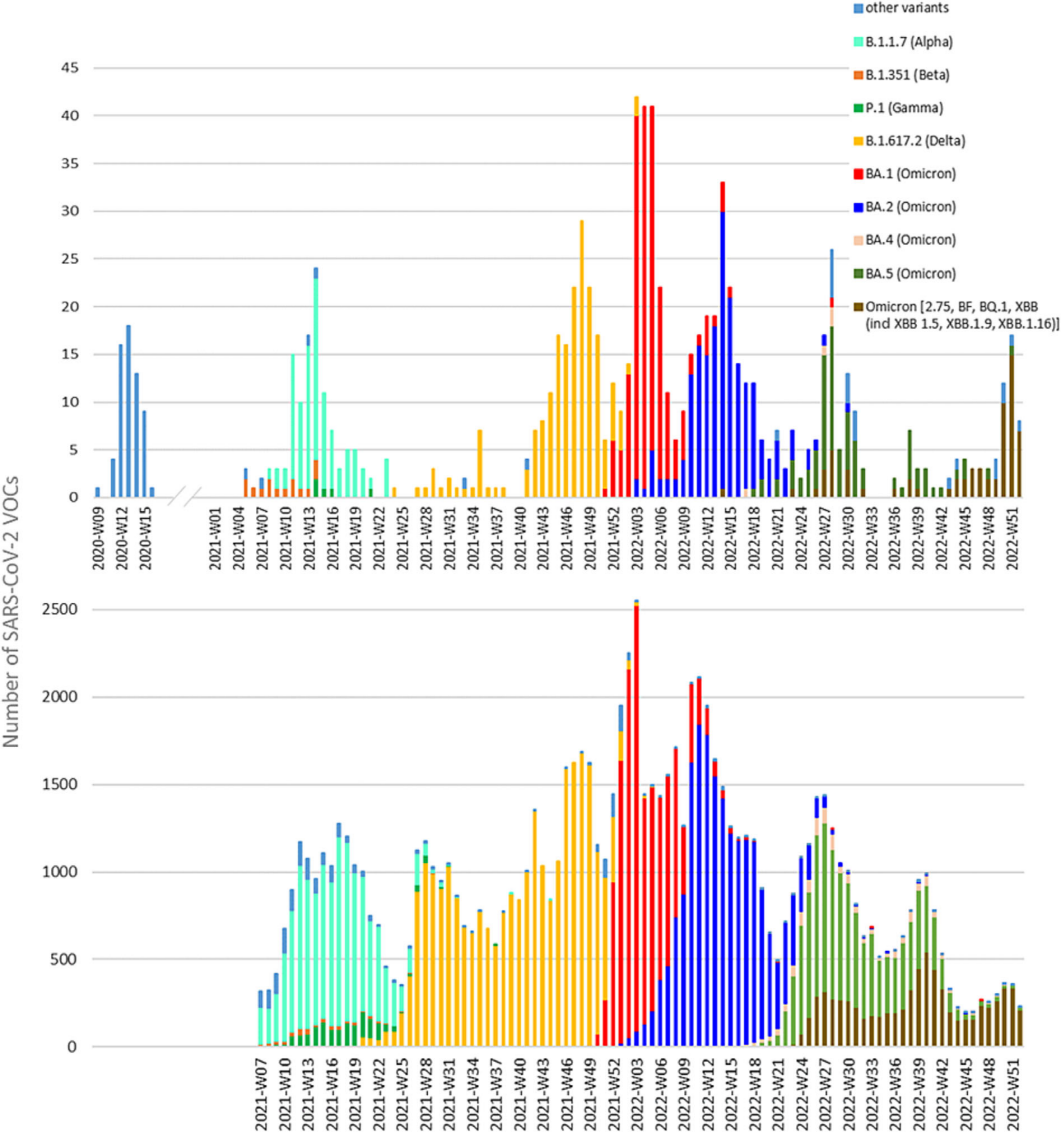
SARS‐CoV‐2 VOC dynamics during the COVID‐19 pandemic registered by the SARI surveillance (upper graph) and the national baseline genomic surveillance (lower graph). Both surveillance systems shows similar patterns. The national baseline genomic surveillance officially started in Week 7 of 2021, whereas SARI surveillance sequencing was retrospectively performed since 2020‐W09. The results of the baseline genomic surveillance are based on a total of 96,251 sequences, whereas those of the SARI surveillance system are based on 964 sequences. Wuhan‐like strains and non‐VOC variants (detected in 2020 by SARI surveillance) are included in “other variants.”

SARS‐CoV‐2 was first detected within the SARI surveillance for a patient sampled on March 1, 2020. This was the same day as the first (non‐travel‐related) detection of the virus in the general population. Since then, out of 5,695 respiratory samples received between March 13, 2020, and December 31, 2022, 1,558 samples tested positive for SARS‐CoV‐2 by qPCR, and 1,103 (71%) had a Ct value that enabled sequencing. In total, 925 samples were sequenced successfully (Supporting Information S1), covering the different SARS‐CoV‐2 waves of the pandemic in Belgium. Each week, 1 to 44 SARS‐CoV‐2 positive SARI samples were sequenced, except for some weeks where none were sequenced. Only the second wave (August 2020 till February 2021) was missed due to a temporary disruption of the SARI surveillance system, which resumed in 2021‐W04. Since the BGS started officially from 2021‐W07, 859 sequences from the SARI surveillance were included in the analysis. Variant trends provided by these 859 sequences from the SARI surveillance were compared with those determined by the BGS including a total of 96,251 sequences from 2021‐W07 until 2022‐W52. Lineage analysis showed a similar pattern to that observed in the national baseline genomic surveillance (Figure 2).

**FIGURE 3:**
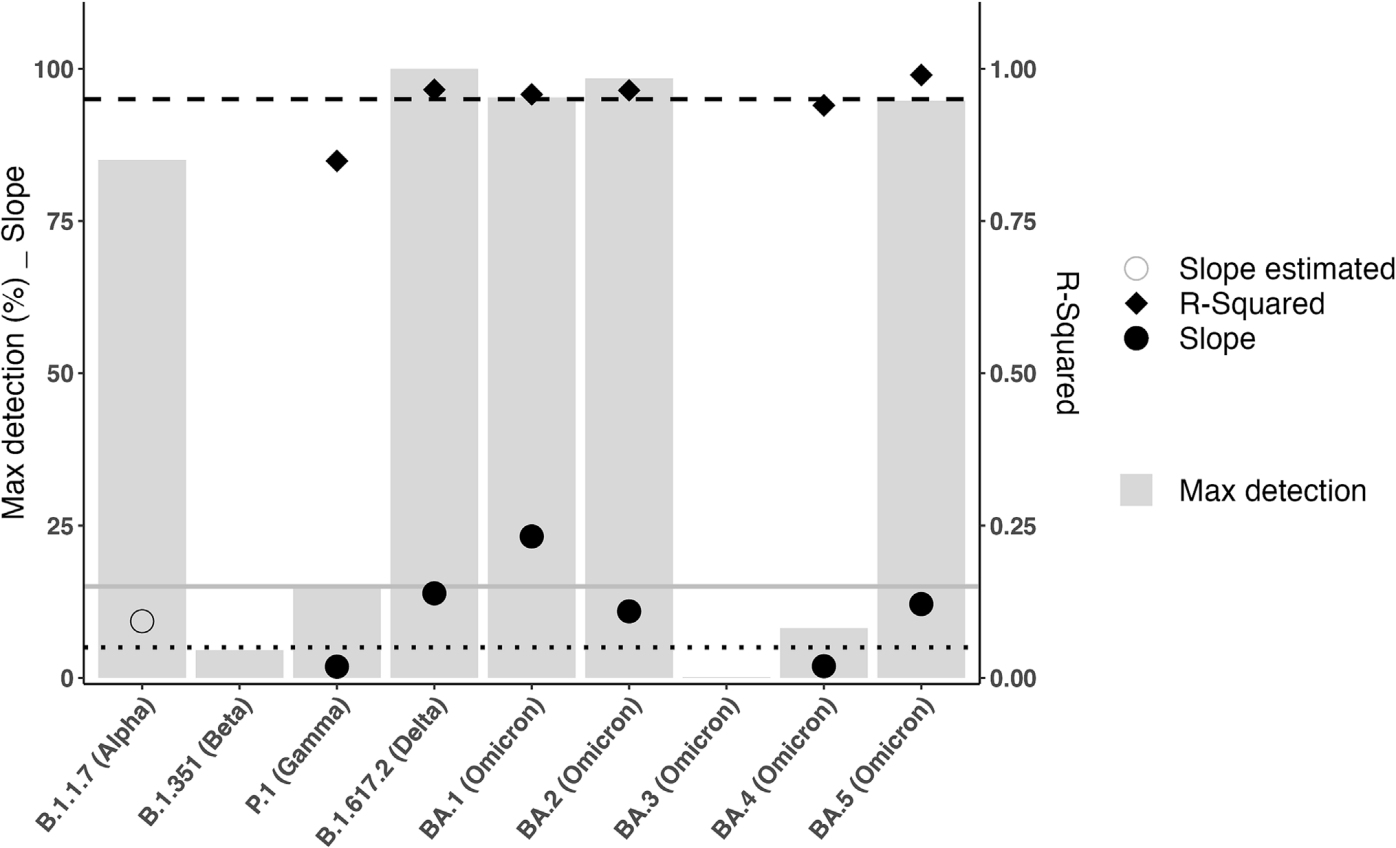
The maximal weekly detection percentages (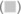) in the national genomic baseline surveillance for each of the different VOCs (Alpha, Beta, Gamma, Delta, Omicron, and its relatives) and the slope (● / ○) and *R* ^2^ (◆) values of linear regression curves for each (see the supporting information) were combined to define an *emerging* variant. A VOC was considered emerging if complying with the following empirical criteria: *R* ^2^ > 0.95 (dashed line), slope > 5 (dotted line), and >15% weekly incidence (full line). Based on this definition, Variants B.1.1.7, B.1.617.2, BA.1, BA.2, and BA.5 are considered emerging VOCs, while P.1, BA.3, and BA.4 did not emerge. A slope could not be calculated for VOC B.1.351, as its epidemic growth was not exponential.

The speed of epidemic growth was evaluated for each VOC using the slope values at the epidemic growth phase in the baseline genomic surveillance and was highest for BA.1 (23.21), followed by B.1.617.2 (13.87) and BA.5 (12.10) having similar slopes, and BA.2 (10.91). P.1 (1.84) and BA.4 (1.92) numbers increased more slowly. For B.1.1.7, the epidemic curve and its epidemic growth started before the registration of the variants in the Sciensano Dashboard. A prediction of the minimal epidemic growth rate was calculated using the first reported Belgian B.1.1.7 sequence in GISAID (2020‐W52) as starting point and its prevalence value in the BGS at the first measurement point 2021‐W07 (65%), resulting in an estimated minimal slope of 9.28 (Supporting Information S3). The low detection period before the epidemic growth phase varied from 2 to 8 weeks, depending on the VOC. Variants B.1.351 and BA.3 never showed an epidemic growth phase (*R* ^2^[BA.3, B.1.351] = undetermined), and for P.1, the regression curve had a poor *R* ^2^ score of 0.90. Except for B.1.617.2, none of the other VOC reached a 100% weekly detection in the BGS sequences, with maximal weekly detection levels of 85.1% (B.1.1.7), 4.6% (B.1.351), 14.9% (P.1), 95.3% (BA.1), 98.5% (BA.2), 0.14% (BA.3), 7.7% (BA.4), and 69.0% (BA.5) for the studied period (Figure 3; Supporting Informations [Link], [Link], [Link], [Link]).

For this study, we defined a variant as emerging within the Belgian population based on the exponential properties (i.e., the slope and value for *R* ^2^) of its weekly frequency detection curve and its weekly calculated detection level within the total number of sequences as calculated from the baseline surveillance genomic data. We have set empirically following criteria: *R* ^2^ > 0.95, slope > 5 and maximal weekly detection level >15%. The *R* ^2^ value of 0.95 is a general accepted value to define a reliable regression model. The slope value has been arbitrary chosen and set at five, as a proof‐of‐concept to express an important increase in VOC numbers (five times) per time unit (1 week). Where ECDC uses a threshold of 10% to indicate the start of the seasonal epidemic of influenza based on sentinel specimens,^45^ we decided to use 15% as threshold for SARS‐CoV‐2 because of the non‐sentinel character of our dataset. Based on this definition, Variants B.1.617.2, BA.1, BA.2, and BA.5 are considered emerging and referred to as major VOCs, whereas the remaining variants (B.1.351, P.1, BA.3, and BA.4) are considered minor and did not successfully circulate (Figure 3). Variant B.1.1.7 is also considered as a major VOC since it fulfills the definition when using the estimated minimal epidemic growth rate as described previously (Supporting Informations S3 and S4).

### 3.2 Description of the observations per VOC in SARI and the comparison to BGS

**FIGURE 4:**
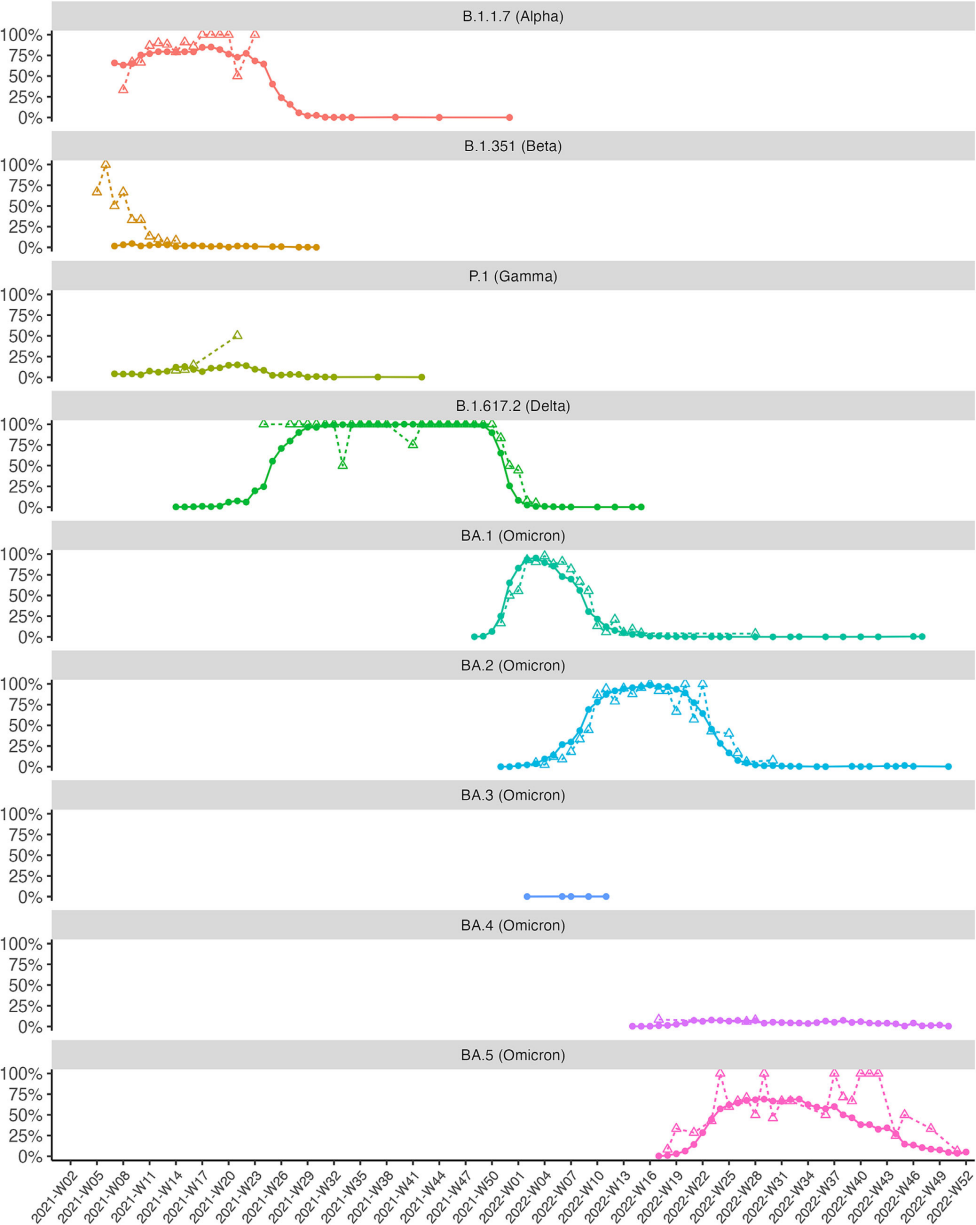
Comparison of SARS‐CoV‐2 genomic surveillance in SARI samples and the Belgian baseline genomic surveillance. The proportions of the five VOC (B.1.1.7 [Alpha], B.1.351 [Beta], P.1 [Gamma], B.1.617.2 [Delta], and BA.1/BA.2/BA.3/BA.4/BA5 [Omicron]) are presented as the percentage of the total number of weekly sequences from January 2021 (Week 7) until December 2022 (Week 52). Dotted lines with empty triangles represent the SARI genomic surveillance sequences; continuous lines with bullets represent the Baseline Genomic surveillance sequences. For readability reasons, Wuhan‐like strains and non‐VOC variants detected in SARI surveillance in 2020 are not included in the figure. Genomic SARI surveillance shows timely detection for each VOC as compared to the baseline surveillance, except for BA.3 (omicron), which was not detected. The observed trends in the proportion of weekly positives of a variant in SARI surveillance are comparable to the waves observed in the baseline surveillance.

The BGS started officially in 2021‐W07, when both VOCs B.1.1.7 and B.1.351 were already circulating at important levels so that no comparison is possible with SARI surveillance samples as for what concerns their first detection. As shown in Figure 4, the primary detection of SARS‐CoV‐2 VOC B.1.1.7 in SARI surveillance occurred in week 2021‐W08, which is 4 weeks after the SARI surveillance resumed in 2021. This is 8 weeks after this variant was first reported in GISAID for Belgium and when its prevalence in the BGS reached 63.2%. This variant was detected in SARI surveillance samples until 2021‐W23. Beta VOC B.1.351 was detected 6 weeks later in the SARI surveillance as compared to first reported sequence in GISAID. Within SARI surveillance, B.1.351 was identified 14 times on a total of 81 sequences (17.3%) during the period 2021‐W05 to 2021‐W14 where it co‐circulated with the more prevalent VOC B.1.1.7 (61 out of 81 sequences, 75.3%). VOC P.1 (Gamma) was first detected in 2021‐W04 in the BGS and could only be picked up 10 weeks later in SARI samples, where its latest detection was in 2021‐W21. During that period (2021‐W14 to 2021‐W21), only 5 out of 60 sequences (8.3%) were found to be P.1 in SARI, with most sequences (52/60, 86.6%) still being B.1.1.7. VOC P.1 did not truly emerge in Belgium, had no exponential increase (*R* ^2^ < 0.95), and reached a maximum weekly detection level of only 14.95% in the BGS. Similarly, the Delta variant B.1.617.2 was also detected with a delay of 10 weeks in SARI surveillance as compared to its first detection in the BGS. Within BGS, this VOC had an extremely long phase of 9 weeks at low detection as compared to the other VOCs, before it spread in the entire population and was detected in SARI samples.

Omicron variants started circulating in 2021‐W48 in the Belgian population, with BA.1 and BA.2 circulating mainly from January to June 2022 and being replaced by BA.4 and BA.5 afterwards. SARI surveillance allowed the detection of BA.2 at its exponential rise, which was 4 weeks after its first detection in the BGS. Omicron variant BA.5, was even detected within a single week after its first detection in the BGS and still being before the exponential rise of the variant. BA.1 and BA.4 were detected 1 week after the variant started increasing in the Belgian population and within 3 weeks after their first detection in BGS. However, Omicron Variant BA.3 was never detected in SARI samples, while it was detected in the BGS but at very low frequencies (max 0.14% among weekly sequences) and without really emerging in the country (Figure 4).

## 4 DISCUSSION

During the SARS‐CoV‐2 pandemic, many countries integrated SARS‐CoV‐2 surveillance in their existing sentinel surveillance systems successfully and allowed those countries to evaluate different epidemiological aspects of this new respiratory pathogen.^9^, ^30^, ^37^, ^46^, ^47^ The Belgian National Influenza Centre also integrated SARS‐CoV‐2 detection in the existing hospital SARI surveillance for influenza.^29^ This allowed sequencing of SARS‐CoV‐2 positive samples and thus variant genomic surveillance during the pandemic, resulting in a total of 964 sequences over the period 2020‐W09 until 2022‐W52. Since the official start of the BGS in 2021‐W07 until end 2022, a total of 96,251 SARS‐CoV‐2 sequences were reported by the COVID‐19 Belgium Genomics Consortium to the Belgium national public health institute, Sciensano.^26^, ^41^ Our data show that SARI surveillance succeeded in detecting the circulating VOCs in a representative manner according to the circulation in the general population using 859 sequences generated for that same period. This study thereby demonstrates the potential of the existing SARI surveillance in the genomic surveillance of SARS‐CoV‐2 VOCs during the pandemic and supports its continued use in a post‐pandemic era.

In SARI surveillance, the B.1.1.7 variant was detected 8 weeks after the first reported sequence in GISAID for Belgium from a returning traveler (EPI_ISL_791333) that was sampled on December 21, 2020 (2020‐W52). At that time, indications for sampling were based on active surveillance and although sequencing efforts were mainly research‐oriented before the generic BGS was established on February 15 to cover the general population, this resulted in over 3,700 available sequences by the end of 2020 and a total of 317 sequences reported on the Sciensano Dashboard for 2021‐W07.^26^, ^41^ Since the sampling in SARI surveillance only resumed in 2021‐W04 and that weekly only one to three SARS‐CoV‐2 positives were appropriate for sequencing between 2021‐W04 to 2021‐W07, sensitivity of SARI surveillance was at that time too low to early detect this VOC although its general prevalence was already significant. Our data show that the other major VOCs B.1.617.2, BA.1, BA.2, and BA.5 were detected in SARI surveillance within the low detection period or with a 1 to 2 weeks delay after the start of their epidemic growth phase as observed in the BGS. For both Omicron derived recombinants BQ1 and BF7, which were considered by WHO as variants of interest (VOI) and variants under monitoring (VUM), respectively, and which are both major variants in the BGS, a timely detection in SARI surveillance was observed as well (results not shown).^41^ The B.1.617.2 variant was detected first in India in December 2020 and was, unlike the major circulating Alpha variant B.1.1.7 at that time, no longer characterized by a S gene dropout in the qPCR used for SARS‐CoV‐2 detection in human samples.^18^, ^48^ Preferential sequencing of samples lacking the S gene drop‐out and from travelers coming from India to capture the introduction of this variant in the country within the BGS was probably common practice.^24^ This could explain both the rapid detection (BGS 2/959 sequences [0,2%] in week 2021‐W14) and the long period of circulation at low levels as observed in the BGS, before its exponential epidemic growth from 2021‐W22 onwards, while not detecting the variant in SARI samples before 2021‐W24. During that period (2021‐W14 to 2021‐W22), only a small number of SARS‐CoV‐2 positive SARI samples were sequenced at the NIC (with a maximum of 24 sequenced samples on a weekly base for that period), and this might have been insufficient to detect this low circulating variant rapidly.

Sequencing in SARI surveillance relies solely on the SARS‐CoV‐2 positivity rate of its samples as it is a case definition‐based sentinel surveillance system. Although this implied that the weekly number of sequences were low, SARI surveillance did succeed to detect the minor variants shortly after their numbers started increasing exponentially in the BGS, except for BA.3, but this VOC never showed an epidemic growth phase. Meanwhile, the BGS aimed at continuously sequencing 5% of all the SARS‐CoV‐2 PCR positive samples in an attempt to comply with the national recommendations (5–10% positives sequenced) and ECDC guidance documents and generated continuously high numbers of sequences.^2^, ^23^, ^24^ This system is however dependent on the total number of tested COVID‐19 cases, which in his turn depends on the testing strategy in Belgium and leads to variations in sensitivity. During epidemic peaks of infections, the imposed threshold of 5% could not be reached consistently.^26^ On the other hand, in periods of lower virus circulation or testing, sequencing coverage reached 14% of positive cases, thereby resulting in a very high sensitivity of the BGS. This sensitivity is especially important for the detection of emerging variants before they reach 1% of the circulating strains as was observed for the B.1.617.2 variant.^2^, ^24^, ^41^ Also, the data gathered from the baseline genomic surveillance allowed the Belgian Sequencing Consortium to conduct or participate to several active surveillance studies concerning phylogenetic analyses, VOC‐associated disease severity assessments and outbreak investigations such as in nursing homes and others.^26^, ^49^, ^50^, ^51^, ^52^, ^53^, ^54^

Regardless the strict non‐pharmaceutical interventions that were implemented in Belgium from March 13, 2020, onwards, the SARS‐CoV‐2 pandemic strongly affected the SARI surveillance system due to overburden of the hospitals which were unable to provide timely samples and patient information. Samples within this study were often retrospectively sequenced, and sampling dates were used to establish the figures in this publication. The pandemic also resulted in a decrease in patient samples sent, with on average 50 samples per week (2020‐W14 to 2022‐W52) as compared to normal influenza seasons (during 10 weeks > 100 samples per week; 4 weeks between 50 and 100 samples per week in 2019–2020 season).^55^ From 2020‐W17 to 2020‐W20 (influenza season 2019–2020), the system was even completely interrupted and sampling for the 2020–2021 influenza season could only start from 2021‐W04 onwards. From that week on, the SARI surveillance has been running continuously, all weeks of the year. Our results show that even with a relatively low number of samples and delay in timing of sequencing, SARI surveillance succeeded in detecting the minor and major VOCs that eventually emerged in Belgium in a timely manner. To be able to detect viral variants during both their endemic or pandemic circulation, a year‐round SARI surveillance, subject however to some organizational improvements, should be envisaged and encouraged. This should ideally involve an increase of the number of the participating hospitals to improve the catchment population and being more representative for the whole country but should also require better resources for sampling and faster sample shipment. Not only pandemic preparedness and response will be strengthened, but also the public health management of the yearly epidemics of a number of respiratory viruses will benefit from this system.^27^, ^28^, ^29^, ^56^ The performance of the SARI surveillance system can be increased by including different pathogen‐specific labs or institutions.

Besides the difference in sequencing strategy, an important difference between both surveillance systems lies in the sampling strategy itself. While the BGS collects SARS‐CoV‐2 positive samples from over 40 diagnostic laboratories, relies on decentralized sequencing, but centralized data repository and analysis, sampling in SARI surveillance is based on six sentinel sites recruiting samples based on the clinical presentation that is limited to patients that fulfill the SARI case definition.^26^, ^28^, ^57^ In the context of SARI surveillance in Belgium, testing and sequencing are centralized in the public health laboratories of Sciensano. As the SARS‐CoV‐2 pandemic evolved, both natural immunity due to virus exposure and induced immunity from vaccination programs increased within the population and conferred protection against severe disease, and it will continue to do so in a post‐pandemic era.^57^, ^58^ Together with the changing characteristics of variants while adapting to its host to evade immune response, SARS‐CoV‐2 will circulate in the general population without necessarily causing hospitalization to the same extent as observed during the SARS‐CoV‐2 pandemic, thereby limiting severe infections more to the high‐risk groups (i.e., people aged >60 years or with underlying health conditions) related to this pathogen.^59^, ^60^, ^61^, ^62^ This could result in a lower detection of variants causing mild disease in the targeted population of the SARI surveillance, while variants causing more severe disease might be overrepresented. However, it will become less important to capture these variants causing mild disease in a timely manner, since specific measures are no longer put in place to protect the general population from these particular variants. Anyway, patients suffering from mild respiratory infections are well covered by the existing ILI (influenza‐like illness) surveillance that involves a network of sentinel general practitioners in Belgium since 1979.^63^ This ILI surveillance could complement the SARI surveillance network, the latter still focusing on variants causing severe respiratory infections. Since both SARI and ILI surveillances sampling are symptom based, they both have the advantage of lowering the risk in biased sample selection which at some times will be the case for the baseline surveillance, where samples with specific characteristics of variants of interest or concern might be prioritized for sequencing to rapidly detect introduction of a variant in the country.^26^, ^28^ Also, as the national testing indications change from testing all suspected COVID‐19 patients to only testing very specific subgroups, the representativeness of a BGS will change over time, while the representativeness of the SARI surveillance remains the same.

Viruses evolve continuously and the BGS aims for the rapid detection of new introductions in the general population and emergence of new variants in near real‐time, in order to inform the local authorities and allow a prompt risk assessment.^3^, ^48^ While immediate launch of biomedical research has shown to be efficient on multiple occasions, public health actions such as travel restrictions and targeted testing and tracing during the pandemic following the detection of a new variant have however not proven to allow a sustained containment.^9^, ^11^, ^12^, ^26^, ^64^ Upon its first detection, a variant is often already circulating at low to moderate levels within the population, so often variant‐specific measures have only limited effect on the further spread.^6^, ^9^, ^64^, ^65^ Also, within risk assessment, hazard assessment not only relies on the virological information of a variant retrieved from testing of viral isolates, but also on epidemiological information retrieved after a variant has been circulating for some time, thereby using the numbers of deaths and hospitalized cases that were registered. SARI surveillance aims to gather information on the circulating strains in human cases suffering from a severe respiratory infection during the endemic, epidemic and/or pandemic circulation of a virus.^32^ Although SARI surveillance is less timely and sensitive compared to the baseline genomic surveillance in its current format, it gives a well‐founded assessment on the disease impact of pathogen circulation in a country. This allows for monitoring of locally circulating viruses for antiviral sensitivity or strain identification for the vaccine composition for the upcoming season, both existing already for influenza and possibly applicable for SARS‐CoV‐2 and other pathogens as well.^9^, ^27^, ^28^, ^29^, ^31^, ^66^, ^67^, ^68^

As suggested by Brito et al.,^21^ a nationwide strategy allowing to sequence at least 0.5% of the positive cases, with a turnaround time (time in days between sample collection and genome submission) of less than 21 days, could be a benchmark for viral pathogens genomic surveillance efforts. This means that to maintain its sensitivity, a national BGS would require a high capacity to continuously sequence these important numbers of samples, and the organization of their transport to the sequencing centers to allow near real‐time surveillance should be guaranteed as well. In a different approach, sentinel surveillance provides an alternative solution to measure the impact of a virus or virus variant in the population using a lower number of sequences.^9^ Moreover, the SARI sampling strategy remains unaffected by changes in testing indications. Another major benefit of using SARI and ILI surveillance in genomic SARS‐CoV‐2 variant detection is the availability of clinical patient information allowing for further analysis on severity and/or vaccine effectiveness using the same data. The clinical information sent alongside with the samples has not been used in the current study since it is out of the scope of this publication. Besides, the respiratory specimens sampled in SARI and ILI surveillance undergo long‐term storage and allow for retrospective analysis, as demonstrated in the current study.

We think that sentinel ILI and SARI surveillance networks constitute a useful tool to monitor the circulation of SARS‐CoV‐2 variants, or other respiratory pathogens, subject however to some organizational and logistical adjustments to achieve this purpose and as described in the ECDC guidelines.^31^

## 5 CONCLUSIONS

In conclusion, our results show that sentinel SARI surveillance can give a valuable reflection of the circulation of the different SARS‐CoV‐2 variants of concern during pandemic and endemic periods, especially for variants related to severe respiratory infections. Sentinel SARI surveillance may represent a relatively cost‐effective and sustainable tool to contribute to genomic surveillance, including in low‐ and middle‐income countries.

To reflect circulation of variants causing mild disease in the general population, complementary analysis of ILI surveillance samples from a network of general practitioners could be considered, which is in line with the ECDC recommendation to use existing, sentinel surveillance systems for genomic surveillance of viral pathogens.

Improved and scalable genomic sentinel surveillance systems at a national level, such as integrated ILI and SARI surveillance networks for respiratory pathogens, should be considered as a valuable and reliable option to strengthen the pandemic preparedness and response. Such systems are important not only for monitoring of SARS‐CoV‐2 but also other respiratory pathogens such as influenza virus and RSV.

## AUTHOR CONTRIBUTIONS

**Sarah Denayer:** Conceptualization; formal analysis; supervision; visualization; writing—original draft; writing—review and editing. **François E. Dufrasne:** Methodology; supervision; writing—original draft; writing—review and editing. **Bert Monsieurs:** Investigation. **Reinout Van Eycken:** Investigation. **Sarah Houben:** Visualization. **Lucie Seyler:** Resources; writing—review and editing. **Thomas Demuyser:** Resources; writing—review and editing. **Els Van Nedervelde:** Resources; writing—review and editing. **Marc Bourgeois:** Resources; writing—review and editing. **Benedicte Delaere:** Resources; writing—review and editing. **Benedicte Lissoir:** Resources; writing—review and editing. **Koen Magerman:** Resources; writing—review and editing. **Door Jouck:** Resources; writing—review and editing. **Catherine Sion:** Resources; writing—review and editing. **Marijke Reynders:** Resources; writing—review and editing. **Evelyn Petit:** Resources; writing—review and editing. **Nicolas Dauby:** Resources; writing—review and editing. **Marc Hainaut:** Resources; writing—review and editing. **Lies Laenen:** Writing—review and editing. **Piet Maes:** Writing—review and editing. **Guy Baele:** Writing—review and editing. **Simon Dellicour:** Writing—review and editing. **Lize Cuypers:** Writing—review and editing. **Emmanuel Andre:** Writing—review and editing. **Simon Couvreur:** Resources. **Ruben Brondeel:** Resources. **Cyril Barbezange:** Writing—review and editing. **Nathalie Bossuyt:** Conceptualization; writing—review and editing. **Steven Van Gucht:** Conceptualization; writing—original draft; writing—review and editing.

## CONFLICT OF INTEREST STATEMENT

The authors declare no conflict of interest.

### PEER REVIEW

The peer review history for this article is available at https://www.webofscience.com/api/gateway/wos/peer-review/10.1111/irv.13202.

## ETHICS STATEMENT

The study was conducted in liaison to the trial Surveillance of severe cases of influenza through a sentinel network of hospitals (B.U.N. 143201215671) and approved by the Commissie Medische Ethiek (O.G. 016) under the case number 2012/310. The Ethics Committee is organized and operates according to ICH‐GCP and its applicable laws and regulations. Patient informed consent (oral or written) is part of B.U.N. 143201215671. Regarding Data Protection Regulation, the SARI surveillance obtained approval of the national sectoral social security and health committee in 2015 with decision 15/043 of June 16, 2015.

## Supporting information


**Data S1.** **Supporting information**: GISAID numbers and references of sequences from SARI surveillance respiratory samples used in this study.

**Data S2.** **Supporting information**: Representation of the calculated regression curves, maximal weekly detection percentages and low circulation phase representation for the five VOC [B.1.1.7 (Alpha), B.1.351 (Beta), P.1 (Gamma), B.1.617.2 (Delta) and BA.1/BA.2/BA.3/BA.4/BA5 (Omicron)] in the national genomic surveillance. The low circulation phase before starting its exponential increase is represented for each VOC with a blue zone. This zone is absent for B.1.1.7 and B.1.351, as these variants were circulating already at low levels before the official start of the genomic baseline surveillance in 2021‐W07. The linear regression curves were calculated from the first week at start of the exponential increase until the maximal weekly value was reached for the VOC. Since BA.3 was only detected at very low levels and no exponential increase was observed, it was not possible to draw a regression curve. The equation and R^2^ values calculated from the graphs are available in Supporting information 4.

**Data S3.** **Supporting information**: B.1.1.7 (Alpha), the minimal slope value was estimated using the first reported Belgian sequence in GISAID (EPI_ISL_791333; 2020‐W52) and its prevalence of 65.93% at the start of the Genomic Baseline Surveillance in 2021‐W07.

**Data S4.** **Supporting information**: Equations and R^2^‐values of the calculated regression curves drawn for each VOC for the five VOC [B.1.1.7 (Alpha), P.1 (Gamma), B.1.617.2 (Delta) and BA.1/BA.2/BA.4/BA5 (Omicron)] in the national baseline genomic surveillance are represented. For B.1.1.7 (Alpha), the minimal slope value was estimated using the first reported Belgian sequence in GISAID (2020‐W52) and its prevalence at the start of the Genomic Baseline Surveillance (2021‐W07) (Supporting information 2). Since VOCs B.1.351 (Beta) and BA.3 were only detected at very low levels and no exponential increase was observed, it was not possible to draw a true regression curve (Supporting information 5). The start and end point used for each of the regression curves are presented. The equation and R^2^ values are used to extract the slope (a‐value in *y = **a**x + b*) as a measure of increase of a variant of concern in the surveillance, and to evaluate the quality of the regression curve. A variant was defined as emerging according to the values for the slope, R^2^ and maximal weekly detection (see figure 3).

**Data S5.** **Supporting information**: Representation of the prevalence of the variants of concern B.1.351 (Beta), BA.3 (Omicron) in the baseline genomic surveillance. Both were only detected at very low levels and no exponential increase was observed.

## Data Availability

The datasets generated and/or analyzed during the current study are publicly accessible in the GISAID repository (available at https://gisaid.org/). The GISAID accession identifiers of the sequences analyzed in this study are provided as part of Supporting Information S1. Graphs with calculations of the regression curves, its slope, and *R* ^2^ for each variant are provided in Supporting Informations [Link], [Link], [Link], [Link].
