# Supplementary material for: Genomic monitoring of SARS‐CoV‐2 variants using sentinel SARI hospital surveillance
Source: Influenza Other Respir Viruses. 2023 Oct 13;17(10):e13202. doi: 10.1111/irv.13202 (PMC10570899; doi:10.1111/irv.13202)

**Supporting information 2**: Representation of the calculated regression curves, maximal weekly detection percentages and low circulation phase representation for the five VOC [B.1.1.7 (Alpha), B.1.351 (Beta), P.1 (Gamma), B.1.617.2 (Delta) and BA.1/BA.2/BA.3/BA.4/BA5 (Omicron)] in the national genomic surveillance. The low circulation phase before starting its exponential increase is represented for each VOC with a blue zone. This zone is absent for B.1.1.7 and B.1.351, as these variants were circulating already at low levels before the official start of the genomic baseline surveillance in 2021-W07. The linear regression curves were calculated from the first week at start of the exponential increase until the maximal weekly value was reached for the VOC. Since BA.3 was only detected at very low levels and no exponential increase was observed, it was not possible to draw a regression curve. The equation and R² values calculated from the graphs are available in Supporting information 4.


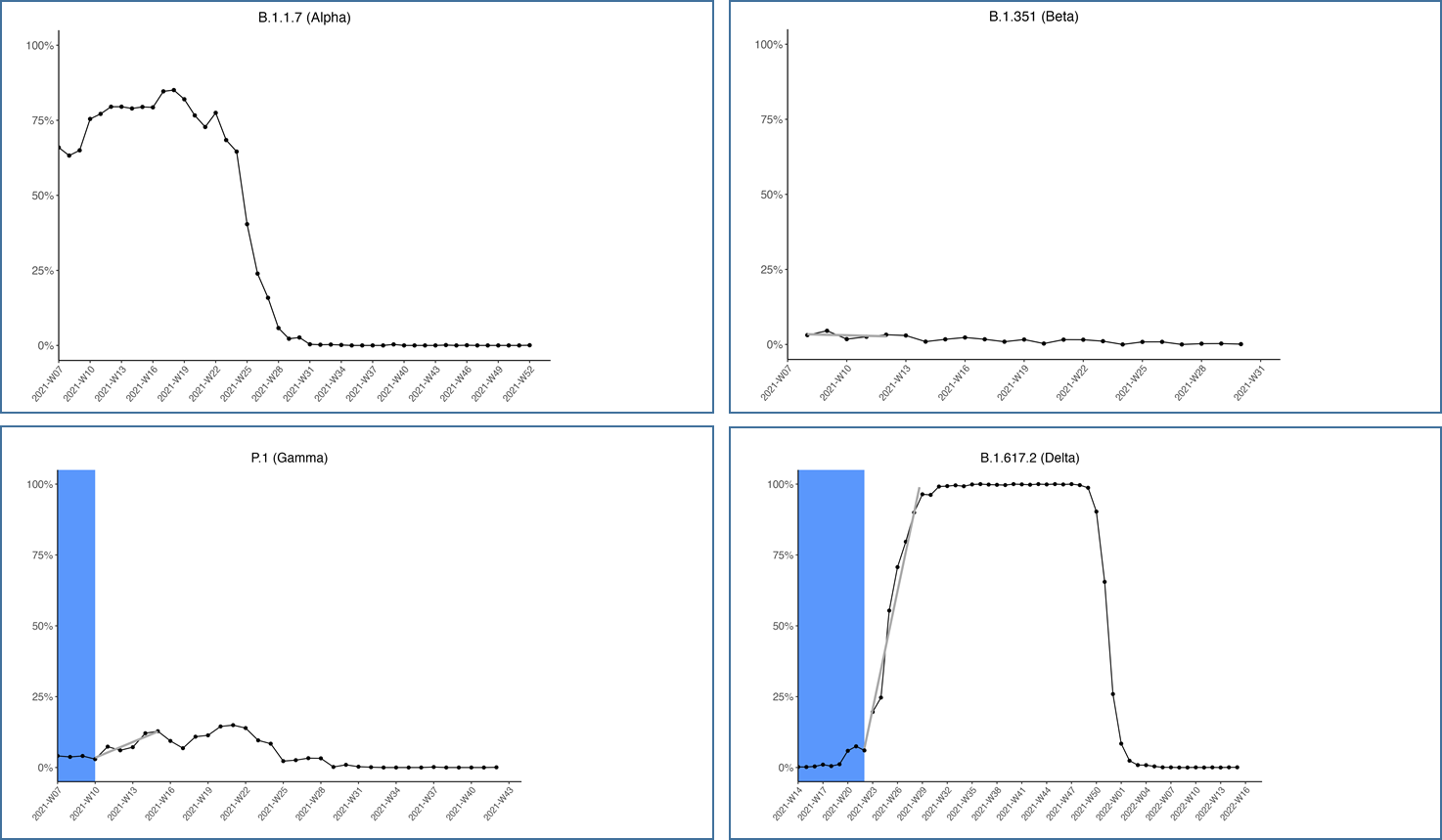


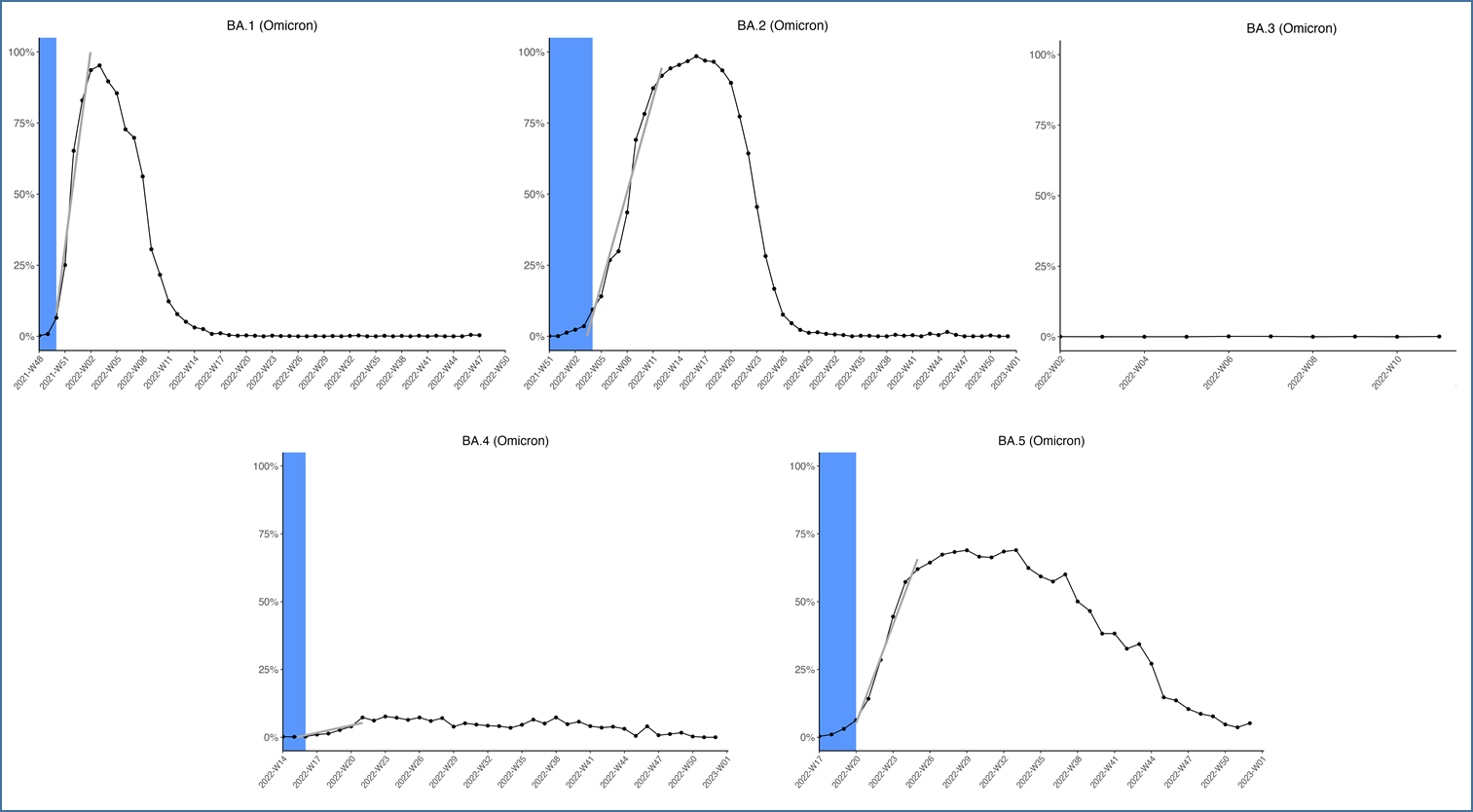

Supplement: Supplementary file 2 — Data S2. Supporting information: Representation of the calculated regression curves, maximal weekly detection percentages and low circulation phase representation for the five VOC [B.1.1.7 (Alpha), B.1.351 (Beta), P.1 (Gamma), B.1.617.2 (Delta) and BA.1/BA.2/BA.3/BA.4/BA5 (Omicron)] in the national genomic surveillance. The low circulation phase before starting its exponential increase is represented for each VOC with a blue zone. This zone is absent for B.1.1.7 and B.1.351, as these variants were circulating already at low levels before the official start of the genomic baseline surveillance in 2021‐W07. The linear regression curves were calculated from the first week at start of the exponential increase until the maximal weekly value was reached for the VOC. Since BA.3 was only detected at very low levels and no exponential increase was observed, it was not possible to draw a regression curve. The equation and R2 values calculated from the graphs are available in Supporting information 4. [file IRV-17-e13202-s003.docx]
