# Supplementary material for: Genomic monitoring of SARS‐CoV‐2 variants using sentinel SARI hospital surveillance
Source: Influenza Other Respir Viruses. 2023 Oct 13;17(10):e13202. doi: 10.1111/irv.13202 (PMC10570899; doi:10.1111/irv.13202)

**Supporting information 3**: B.1.1.7 (Alpha), the minimal slope value was estimated using the first reported Belgian sequence in GISAID (EPI_ISL_791333; 2020-W52) and its prevalence of 65.93% at the start of the Genomic Baseline Surveillance in 2021-W07.


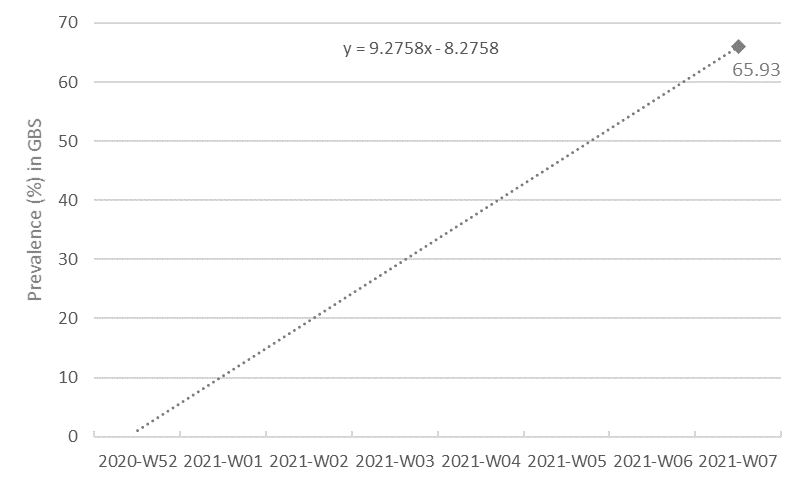

Supplement: Supplementary file 3 — Data S3. Supporting information: B.1.1.7 (Alpha), the minimal slope value was estimated using the first reported Belgian sequence in GISAID (EPI_ISL_791333; 2020‐W52) and its prevalence of 65.93% at the start of the Genomic Baseline Surveillance in 2021‐W07. [file IRV-17-e13202-s004.docx]
