# Supplementary material for: Genomic monitoring of SARS‐CoV‐2 variants using sentinel SARI hospital surveillance
Source: Influenza Other Respir Viruses. 2023 Oct 13;17(10):e13202. doi: 10.1111/irv.13202 (PMC10570899; doi:10.1111/irv.13202)
Supplement: Supplementary file 4 — Data S4. Supporting information: Equations and R2‐values of the calculated regression curves drawn for each VOC for the five VOC [B.1.1.7 (Alpha), P.1 (Gamma), B.1.617.2 (Delta) and BA.1/BA.2/BA.4/BA5 (Omicron)] in the national baseline genomic surveillance are represented. For B.1.1.7 (Alpha), the minimal slope value was estimated using the first reported Belgian sequence in GISAID (2020‐W52) and its prevalence at the start of the Genomic Baseline Surveillance (2021‐W07) (Supporting information 2). Since VOCs B.1.351 (Beta) and BA.3 were only detected at very low levels and no exponential increase was observed, it was not possible to draw a true regression curve (Supporting information 5). The start and end point used for each of the regression curves are presented. The equation and R2 values are used to extract the slope (a‐value in y = ax + b) as a measure of increase of a variant of concern in the surveillance, and to evaluate the quality of the regression curve. A variant was defined as emerging according to the values for the slope, R2 and maximal weekly detection (see figure 3). [file IRV-17-e13202-s002.docx]

**Supporting information 4**: Equations and R²-values of the calculated regression curves drawn for each VOC for the five VOC [B.1.1.7 (Alpha), P.1 (Gamma), B.1.617.2 (Delta) and BA.1/BA.2/BA.4/BA5 (Omicron)] in the national baseline genomic surveillance are represented. For B.1.1.7 (Alpha), the minimal slope value was estimated using the first reported Belgian sequence in GISAID (2020-W52) and its prevalence at the start of the Genomic Baseline Surveillance (2021-W07) (Supporting information 3). Since VOCs B.1.351 (Beta) and BA.3 were only detected at very low levels and no exponential increase was observed, it was not possible to draw a true regression curve (Supporting information 5). The start and end point used for each of the regression curves are presented. The equation and R² values are used to extract the slope (a-value in *y =* ***a****x + b*) as a measure of increase of a variant of concern in the surveillance, and to evaluate the quality of the regression curve. A variant was defined as emerging according to the values for the slope, R² and maximal weekly detection (see figure 3).

| **Variant** | **Equation** | **R²-value** | **Starting point** | **Endpoint** |
| --- | --- | --- | --- | --- |
| **B 1.1.7 (Alpha)** | y = 9.28 x - 8.28 | *Estimated values, see Supporting information 3* | | |
| **B.1.351 (Beta)** | *See Supporting information 5* | | | |
| **P.1 (Gamma)** | y = 1.84 x + 1.62 | 0.8487 | 2021-W10  (8-03-2021) | 2021-W15  (12-04-2021) |
| **B.1.617.2 (Delta)** | y = 13.87 x – 7.09 | 0.9657 | 2021-W22  (31-05-2021) | 2021-W29  (19-07-2021) |
| **BA.1 (Omicron)** | y = 23.21 x -14.97 | 0.9580 | 2021-W50  (13-12-2021) | 2022-W02  (10-01-2022) |
| **BA.2 (Omicron)** | y = 10.91 x – 14.64 | 0.9647 | 2022-W03  (17-01-2022) | 2022-W12  (21-03-2022) |
| **BA.3 (Omicron)** | *See Supporting information 5* | | | |
| **BA.4 (Omicron)** | y = 1.92 x – 0.99 | 0.94 | 2022-W16  (18-04-2022) | 2022-W21  (23-05-2022) |
| **BA.5 (Omicron)** | y = 12.10 x -6.90 | 0.9819 | 2022-W20  (16-05-2022) | 2022-W25  (20-06-2022) |
