# Supplementary material for: Genomic monitoring of SARS‐CoV‐2 variants using sentinel SARI hospital surveillance
Source: Influenza Other Respir Viruses. 2023 Oct 13;17(10):e13202. doi: 10.1111/irv.13202 (PMC10570899; doi:10.1111/irv.13202)

**Supporting information 5**: Representation of the prevalence of the variants of concern B.1.351 (Beta), BA.3 (Omicron) in the baseline genomic surveillance. Both were only detected at very low levels and no exponential increase was observed.


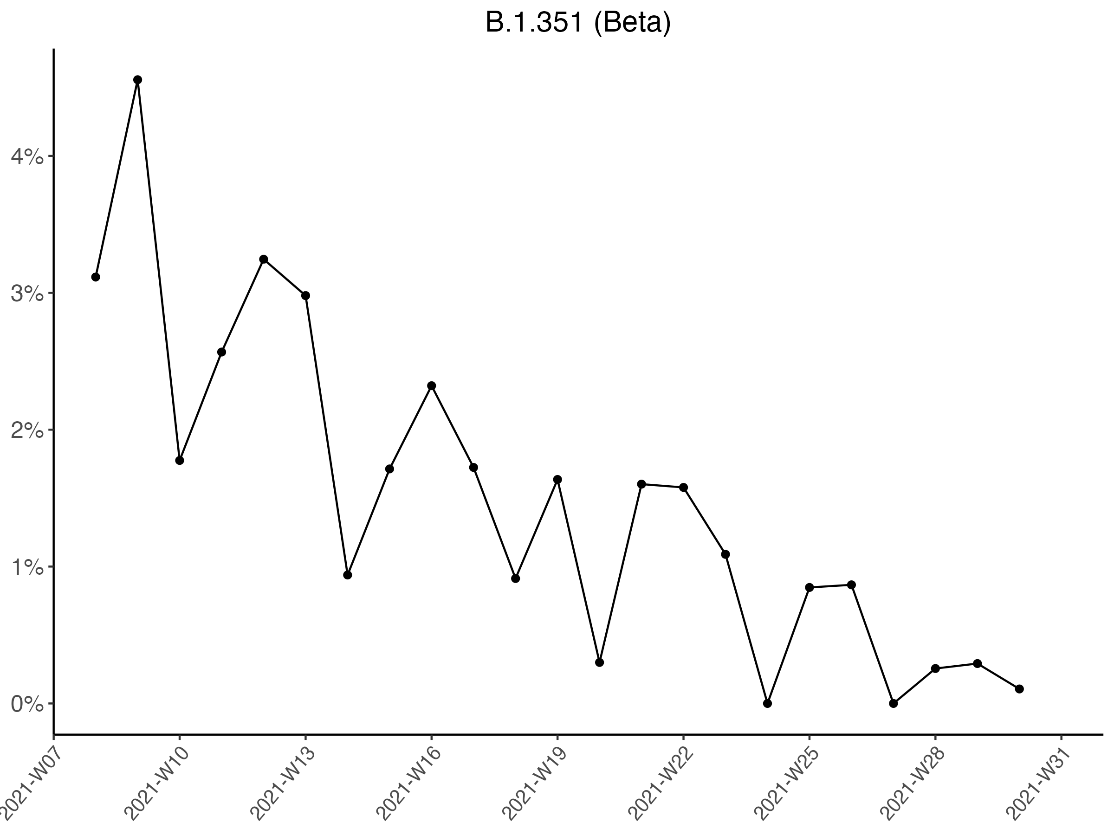


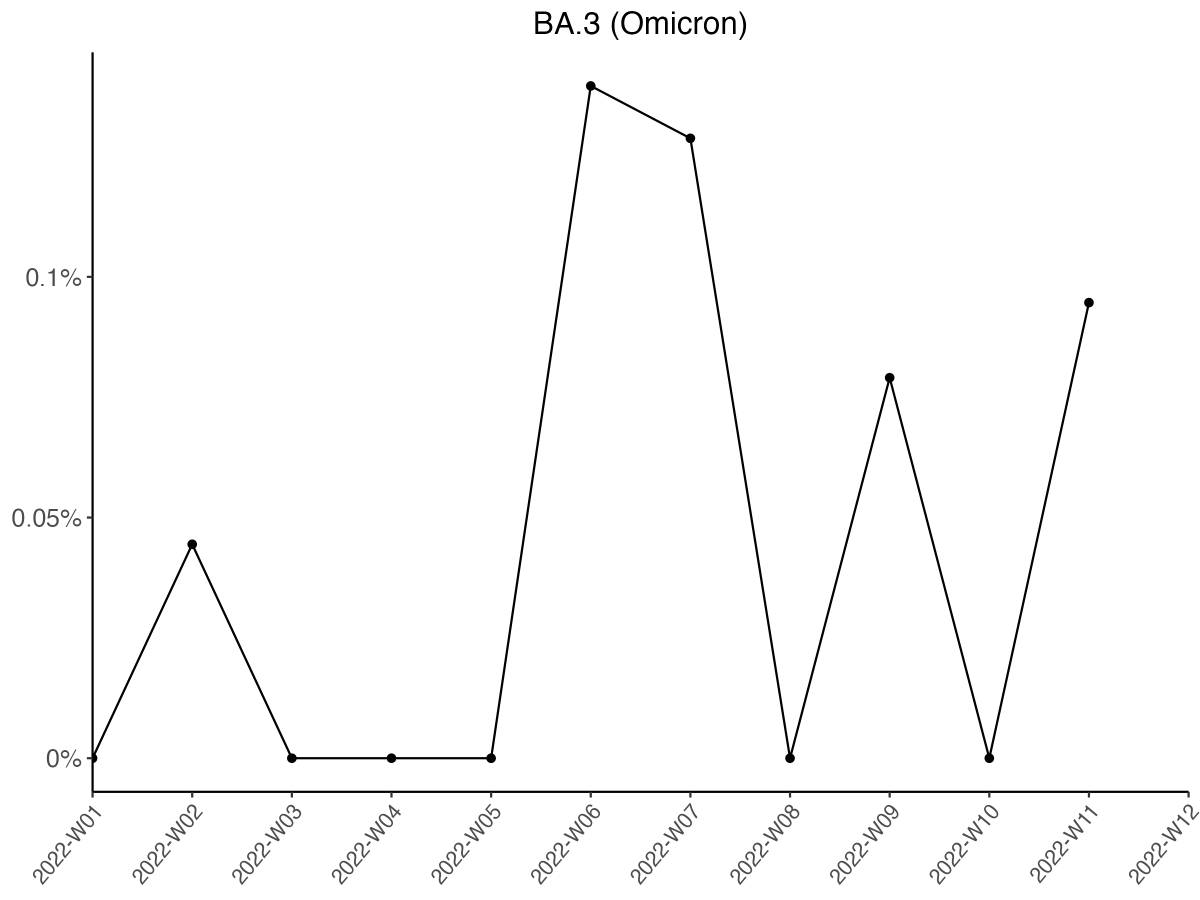

Supplement: Supplementary file 5 — Data S5. Supporting information: Representation of the prevalence of the variants of concern B.1.351 (Beta), BA.3 (Omicron) in the baseline genomic surveillance. Both were only detected at very low levels and no exponential increase was observed. [file IRV-17-e13202-s001.docx]
